# Oxyhydroxide-Coated PEO–Treated Mg Alloy for Enhanced Corrosion Resistance and Bone Regeneration

**DOI:** 10.3390/jfb13020050

**Published:** 2022-05-01

**Authors:** Juning Xie, Shi Cheng, Guoqing Zhong, Ruixiang Zhou, Chi Zhang, Yue He, Yu Zhang, Feng Peng

**Affiliations:** 1 School of Medicine, South China University of Technology, Guangzhou 510006, China; juningxie@163.com (J.X.); raul86@126.com (Y.H.); 2 Medical Research Center, Department of Orthopedics, Guangdong Provincial People’s Hospital, Guangdong Academy of Medical Sciences, Guangzhou 510080, China; chengshi@gdph.org.cn (S.C.); gqzhong@foxmail.com (G.Z.); 15616228413@163.com (R.Z.); zhangchi@gdph.org.cn (C.Z.); 3 Medical College, Shantou University, Shantou 515041, China; 4 Medical Research Center, Jinzhou Medical University, Jinzhou 121001, China

**Keywords:** biomedical magnesium alloy, corrosion resistance, bone repair, surface modification

## Abstract

Plasma electrolytic oxidation (PEO) is widely used as a surface modification method to enhance the corrosion resistance of Mg alloy, the most likely applied biodegradable material used in orthopedic implants. However, the pores and cracks easily formed on the PEO surface are unfavorable for long-term corrosion resistance. In this study, to solve this problem, we used simple immersion processes to construct Mn and Fe oxyhydroxide duplex layers on the PEO-treated AZ31 (PEO–Mn/Fe). As control groups, single Mn and Fe oxyhydroxide layers were also fabricated on PEO (denoted as PEO–Mn and PEO–Fe, respectively). PEO–Mn showed a similar porous morphology to the PEO sample. However, the PEO–Fe and PEO–Mn/Fe films completely sealed the pores on the PEO surfaces, and no cracks were observed even after the samples were immersed in water for 7 days. Compared with PEO, PEO–Mn, and PEO–Fe, PEO–Mn/Fe exhibited a significantly lower self-corrosion current, suggesting better corrosion resistance. In vitro C3H10T1/2 cell culture showed that PEO–Fe/Mn promoted the best cell growth, alkaline phosphatase activity, and bone-related gene expression. Furthermore, the rat femur implantation experiment showed that PEO–Fe/Mn–coated Mg showed the best bone regeneration and osteointegration abilities. Owing to enhanced corrosion resistance and osteogenesis, the PEO–Fe/Mn film on Mg alloy is promising for orthopedic applications.

## 1. Introduction

To date, the most widely and successfully used clinical orthopedic biomaterials are metals such as Ti, Ti alloys, Co–Cr alloy, and 316 stainless steel, owing to their good mechanical properties and biocompatibility [1,2,3]. However, long-term clinical studies have revealed several drawbacks to these non-degradable metals, including stress-shielding effects, host response, and inflammation caused by elastic modulus mismatch, permanent implantation, and wear particles, respectively [4,5,6]. To avoid these drawbacks, researchers have focused on biodegradable Mg, which is considered a next-generation biomedical metal; its elastic modulus is close to that of natural bone (41 vs. 7 to 30 GPa) [7], its low standard electrode potential (−2.37 V vs. normal hydrogen electrode) leads to complete degradation in fluids [8], and its mechanical strength is suitable for orthopedic implants [9,10].

However, Mg-based orthopedic implants are not applied on a large scale, mainly due to their rapid degradation [11,12,13], which not only results in the reduction of mechanical strength but also causes the accumulation of excessive OH^−^ and H_2_. The former might lead to implantation failure, and the latter inhibits bone regeneration. Therefore, enhancing the corrosion resistance of Mg is of great importance for its orthopedic applications.

Surface modification is effective in improving the corrosion resistance of Mg. Commonly used technologies for surface modification of Mg include plasma electrolytic oxidation (PEO), hydrothermal treatment, spray coating, fluoride treatment, etc. [14,15,16]. Among these technologies, PEO coating, performed under high voltage, is widely accepted in the industry. The main component of PEO coating is metal oxide; therefore, it can provide favorable corrosion protection for the substrate. Moreover, high temperatures (over 2000 °C) on the substrate surface can melt the coating and result in strong binding forces between the coating and substrate [17,18]. Numerous studies have treated Mg with PEO and investigated its orthopedic applications [19,20]. Rendenbach et al. modified WE43 Mg alloy with PEO treatment and found that the PEO-treated Mg alloy showed enhanced corrosion resistance and osteointegration upon implantation in Gottingen miniature pigs [21]. However, the dielectric breakdown effect caused pore formation and cracks easily formed and spread over the PEO coating, which is unfavorable for long-term corrosion resistance. A study by Fischerauer et al. revealed that PEO-coated ZX50 Mg alloy vanished completely after 12–16 weeks of implantation in rat femurs [22]. Therefore, a manner by which pores and cracks on the PEO coating can be avoided remains a challenge for PEO-treated Mg applications in orthopedic implants.

Mn and Fe are trace elements that participate in numerous physiological reactions. In addition, both the ions can be adsorbed on alkaline surfaces to form hydroxide. Therefore, in this study, we first used a simple immersing method to fabricate MnOOH and FeOOH coatings on Mg alloy. Interestingly, we found that MnOOH did not change the surface morphology of the PEO coating. However, for the FeOOH film, a layer of nano-sheet-like structures formed on the PEO coating and totally sealed the pores. Hence, to obtain a more protective oxyhydroxide coating on the Mg alloy, we designed and fabricated a duplex Mn/Fe oxyhydroxide (with an inner MnOOH layer and an outer FeOOH layer) on the top of the PEO coating. The corrosion resistance of the newly designed film was investigated. Moreover, the in vitro and in vivo osteogenesis performances of the coated Mg alloy were studied via rat bone marrow stem cell (rBMSCs) cultivation and bone implantation experiments, respectively.

## 2. Materials and Methods

### 2.1. Sample Preparation and Characterization

AZ31 magnesium alloy sheets (with 3% Al, 0.8% Zn, 0.4% Mn, and the balance Mg) were purchased from Suzhou plain metal materials Co., Ltd. (Suzhou, China) and cut into pieces of 10 mm in diameter and 2 mm in length for in vitro tests and pieces of 2 mm in diameter and 8 mm in length for in vivo tests. The AZ31 specimens were ground with 800# silicon carbide abrasive paper and then ultrasonically cleaned in ethanol. The PEO process was conducted in the electrolyte containing 10 g/L C_3_H_7_Na_2_O_6_P and 12.5 g/L KOH. The constant current, frequency, duty cycle, and stop voltage were 0.8 A, 1000 Hz, 10%, and 340 V, respectively. The PEO-treated specimens were then immersed in 12 g/L of MnCl_2_·4H_2_O for 9 h, and the obtained samples were labelled as PEO–Mn. Similarly, after immersion in 2 g/L of FeCl_2_·4H_2_O for 4 h, the samples were denoted as PEO–Fe. The specimens first immersed in 12 g/L of MnCl_2_·4H_2_O for 9 h and then in 2 g/L of FeCl_2_·4H_2_O for 4 h were denoted as PEO–Mn/Fe. The surface views were determined using scanning electron microscopy (SEM; S-3400N, HITACHI, Tokyo, Japan) with a working voltage of 15 kV and an emission current of 0.16 mA. The phase compositions were determined using X-ray diffraction (XRD; D2PHASE, Bruker, Billerica, MA, USA) with a scanning rate of 5°/min and a step size of 0.02°. Element compositions were determined using energy dispersive spectrometry (EDS; IXRF-550i, IXRF SYSTEMS, Austin, TX, USA) with a working voltage of 15 kV and a detection time of 15 min. X-ray photoelectron spectroscopy (XPS; RBD upgraded PHI-5000C ESCA system, Perkin Elmer, Waltham, MA, USA) was conducted with an energy step size of 1 eV, a working voltage of 12 kV, and a filament current of 6 mA.

### 2.2. Corrosion Evaluation

A potentiodynamic polarization test was conducted in phosphate buffered saline (PBS) using an electrochemical analyzer (CHI760C, Shanghai, China). The samples were kept in PBS to obtain a stable open circuit potential and the potentiodynamic polarization test was performed from −2 to 0 V at a scan rate of 10 mV/s.

The samples were placed in a 24-well plate and then 1 mL of α minimum essential medium culture medium (α-MEM) was added to each well. After incubation at 37 °C for 7 days, the samples were collected and rinsed with ultrapure water. The corrosion morphology of the samples was observed using SEM. Moreover, all the culture medium changed from reddish to dark red, indicating that the pH value of culture medium increased.

### 2.3. Live/Dead Staining

C3H10T1/2 cells were purchased from the Type Culture Collection of the Chinese Academy of Sciences and cultured in Modified Eagle’s medium (MEM; Gibco, Waltham, MA, USA) containing 10% FBS, 2 mM L-glutamine (Sigma Aldrich, Missouri, MO, USA) and 1% sodium pyruvate (Leagene Biotechnology, Beijing, China). The conditions of the cell incubator were 37 °C, 5% CO_2_, and 95% humidity [23]. All the in vitro cell experiments were conducted using C3H10T1/2.

Before all experiments, the materials were sterilized by ethylene oxide gas and maintained for 7 days at room temperature to clear remnants. C3H10T1/2 cells were used to evaluate in vitro biocompatibility of the samples. The cells were seeded on the surface of each sample at a density of 5 × 10^4^ cells/mL and cultured for 3 days. Thereafter, the samples were rinsed with PBS and 500 μL of fetal bovine serum–free medium containing calcein-AM (2 μM) and propidium iodide (5 μM) was added to each well, followed by incubation in the dark for 15 min. Ultimately, the samples were rinsed with PBS and the live or dead cells were observed using a fluorescence microscope (Olympus IX 71, Olympus, Tokyo, Japan) with an excitation wavelength of 490 nm for living cells and an excitation wavelength of 545 nm for dead cells.

### 2.4. Cell Proliferation

The cells (5 × 10^4^ cells/mL) were seeded on the sample surfaces and cultured for 1, 3, and 5 days. At each time point, the samples were rinsed with PBS and moved to a new 24-well plate. Immediately, 0.5 mL of AlamarBlue assay (AbD Serotec Ltd., Kidlington, UK; diluted 10-fold with culture medium was added to each well and cultured for another 2 h. Soon after that, 100 μL culture medium from each well was transferred to a black 96-well plate and measured using an enzyme-labeling instrument (BIO-TEK, ELX 800) with an absorption wavelength of 560 nm and a scattering wavelength of 590 nm).

### 2.5. Alkaline Phosphatase (ALP) Activity Assay

The samples were immersed in culture medium (1.25 cm^2^/mL) for 24 h and the extracts were collected for osteogenic differentiation evaluation assays. The cells (5 × 10^4^ cells/mL) were seeded in a 24-well plate and cultured with extracts from different samples supplied with 10 mM β-glycerophosphate, 100 nM dexamethasone, and 50 mM ascorbate and glutamine for 3 and 7 days. At the predetermined time, the BCIP/NBT ALP Color Development Kit (Beyotime, Shanghai, China) was used to stain ALP in the cells in line with the instructions of manufacturer. For quantitative detection, the intracellular ALP activity was quantitated using Alkaline Phosphatase Kit (Beyotime, Shanghai, China) and the total protein was measured using BCA protein quantitation kit (Themo, Waltham, MA, USA). The ALP activity was normalized with total protein content.

### 2.6. Quantitative Real Time Polymerase Chain Reaction (qRT-PCR) Assay

The cells were cultured as described in Section 2.5. Total cellular RNA in each well was collected using Total RNA Kit I (Omega R6834-01, Omega, Guangzhou, China) following the manufacturer’s instructions and the concentration of acquired RNA was measured using a NanoDrop^TM^ 2000 spectrophotometer (ThermoFisher, Waltham, MA, USA). The RNA from each group was then reverse transcribed into complementary DNA (cDNA) using TransScript II All-in-one Fist-Strand cDNA Synthesis SuperMix. The cDNA was then amplified and analyzed by qRT-PCR (TransGen Biotech, Beijing, China) with TransStart Green qPCR SuperMix (TransGen Bioteh, Beijing, China) and primers. The relative expressions of osteoblastic differentiation-related genes, including ALP, osteopontin (OPN), RUNX family transcription factor 2 (RUNX2), collagen-I (COL-I), and osteocalcin (OCN) were quantified using the cycle threshold value and 2^−ΔΔ^CT method. The expression of housekeeping gene glyceraldehyde-3-phosphate dehydrogenase (GAPDH) was used as an endogenous control for normalization. PCR primers sequences are provided in the Table S1.

### 2.7. Bone Implantation Evaluation

All the procedures of animal experiments were performed in accordance with the Guidelines for Care and Use of Laboratory Animals of South China University of Technology and approved by the Animal Ethics Committee of Guangdong Provincial People’s Hospital (KY2020-018-01-01).

Twelve Sprague–Dawley rats (male, 250–300 g) were purchased from Hunan SJA Laboratory Animal Co., Ltd. (Hunan, China) and randomly divided into four groups. After the skin on the bilateral legs was disinfected and lanced, a 2 mm hand-operated drill was used to create a cylindrical hole in the trochlear groove of the femur to reach the marrow cavity, and the materials (2 mm in diameter and 8 mm in length) were implanted in a direction parallel to the longitudinal axis of the femur. The muscles and skin were carefully sutured. All rats were intraperitoneally injected with penicillin for 3 days for protection from postoperative infection. Thereafter, all rats were euthanatized with an overdose injection of pentobarbital sodium at 8 weeks post-surgery, and the bilateral femurs were collected for micro-computed tomography (micro-CT) scanning. The femurs were embedded and cut into sections. The sections were ground, polished, and stained with van Gieson’s (VG) solution. Two femurs for each group were used for VG staining and two random regions for each stained section were pictured. In total, four random regions for each group were pictured to evaluate the area of newly formed bone (the red-stained area) and the distance between the newly formed bone layer and the implant.

### 2.8. Statistical Analysis

Data are presented as the mean ± standard deviation. Differences among groups were analyzed by two-way analysis of variance followed by Tukey’s post hoc test using the GraphPad Prism 8.3.0 (GraphPad Software; San Diego, CA, USA). Statistical significance was set at *p* < 0.05.

## 3. Results and Discussion

**Figure 1:**
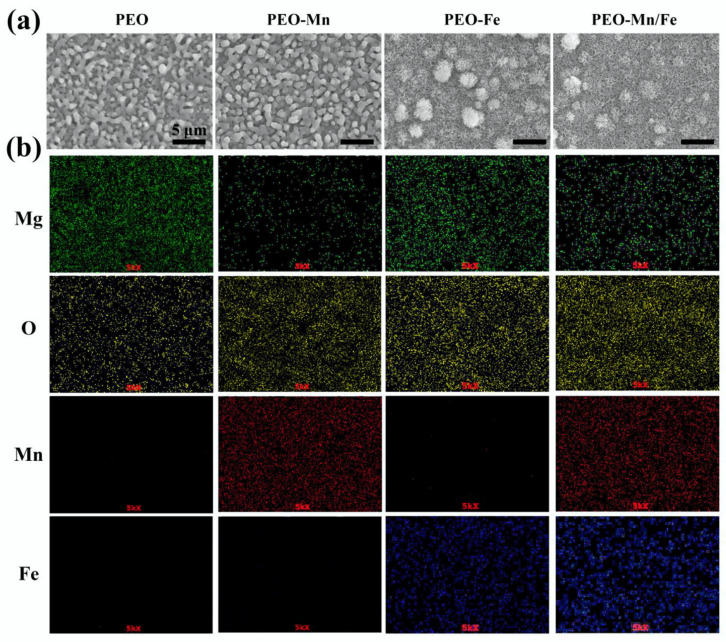
Surface views (**a**) and element distribution (**b**) of PEO, PEO–Mn, PEO–Fe, and PEO–Mn/Fe samples.

The surface views and colors of the various samples are shown in Figure 1a and Figure S1, respectively. PEO samples exhibited a porous structure, the typical morphology of the PEO-treated surface [24,25]. After immersion in MnCl_2_ solution, the surface view of the PEO-Mn sample was similar to that of the PEO sample; however, the grey color changed to black. Upon immersing the PEO-treated sample in FeCl_2_ solution, a layer of nanoflower-like structures which completely sealed the pores was formed. The color of the PEO-Fe sample was dark brown. The surface view of the PEO–Mn/Fe sample showed nanoflower-like structures and was black in color. Figure 1b shows corresponding element distributions of the coated samples. Mn and Fe were uniformly distributed on the PEO–Mn and PEO–Fe samples, respectively. Both Mn and Fe were uniformly distributed on the surface of PEO-Mn/Fe sample. Although no difference was observed on the SEM images of the PEO and PEO–Mn samples, element composition and surface color analysis confirmed that a Mn layer was formed on top of the PEO layer for the PEO–Mn sample. Moreover, the cross-sectional views of the PEO–Fe and PEO–Mn/Fe samples showed a significant layer formed on top of the PEO layer, which is consistent with the surface morphology results in Figure 1a.

**Figure 2:**
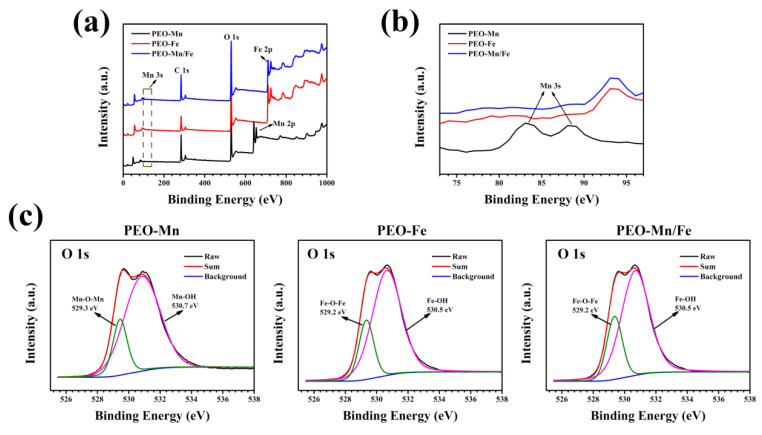
Full XPS spectra (**a**) and enlarged spectra (**b**) of PEO–Mn, PEO–Fe, and PEO–Mn/Fe samples. High-resolution XPS spectra of O 1 s of PEO–Mn, PEO–Fe, and PEO–Mn/Fe samples (**c**).

**Figure 3:**
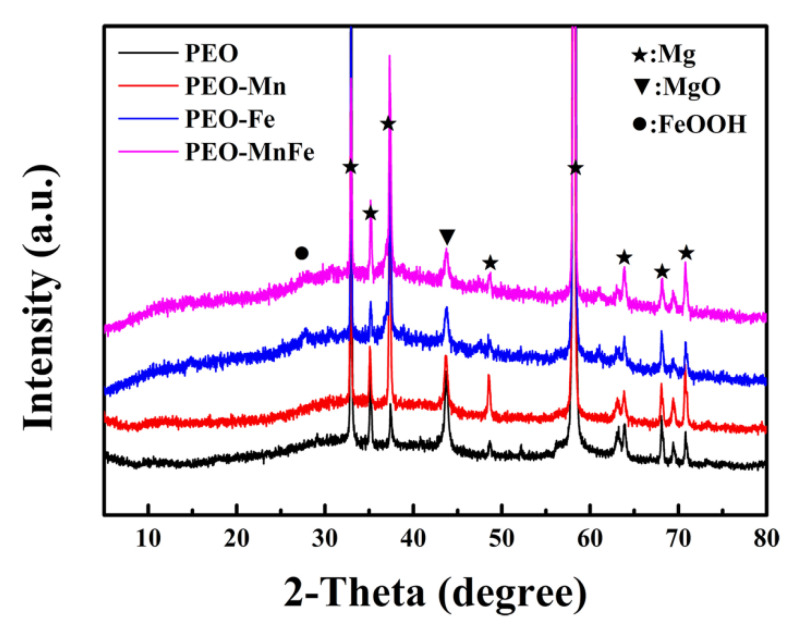
XRD patterns of PEO, PEO–Mn, PEO–Fe, and PEO–Mn/Fe samples.

To analyze the phase compositions of the films, XPS and XRD were performed. As shown in Figure 2a, C, O, and Mn were detected in the full XPS spectrum of the PEO–Mn sample, whereas C, O, and Fe were detected in the PEO–Fe and PEO–Mn/Fe samples, further confirming the element compositions of the films. The Mn oxidation state was calculated from the following formula: △E = 7.88–0.85n (2 ≤ n ≤ 4), where △E is the separation energy of Mn 3s, and n represents the valence state of Mn [26,27,28]. As shown in Figure 2b, the △E value is approximately 0.53 and hence the calculated n value is approximately 3, which corresponds with the valence state of Mn in MnOOH. High-resolution spectra of O 1s are shown in Figure 2c. O 1s of the PEO–Mn spectrum can be divided into two peaks centered at 529.3 and 530.7 eV, which correspond with the structures of Mn–O–Mn and Mn–OH, respectively. This further confirmed that the outer layer of PEO–Mn sample was MnOOH. Moreover, the PEO–Fe and PEO–Mn/Fe O1s spectra can be fitted into two peaks centered at 529.2 and 530.5 eV, which attributed to Fe–O–Fe and Fe–OH structures. This confirmed that the outer layer of both samples was FeOOH [29,30,31]. Figure 3 shows the XRD patterns of various samples. Obvious peaks at 44° representing the MgO phase were detected for all samples. Minute peaks were observed at 27° for the PEO–Fe and PEO–Mn/Fe samples, which indicated the formation of FeOOH. Barring Mg and MgO peaks, no other characteristic peaks were observed in the PEO–Mn group, possibly because the MnOOH film was amorphous or too thin for detection, as shown in Figure S2, no obvious MnOOH layer was observed on the cross section of PEO–Mn sample.

Both the formation of MnOOH and FeOOH can be ascribed to an alkaline-microenvironment-induced self-assembly process, as described in our previous studies [28,31]. Briefly, MgO, the main component of PEO film, is an alkaline substance that produces OH^-^ near the coating. Generated OH^-^ ions react with M^2+^ ions (M represents Fe and Mn) to form unstable M(OH)_2_. Finally, the M(OH)_2_ layers are oxidized by oxygen in the solution and MOOH is generated.

**Figure 4:**
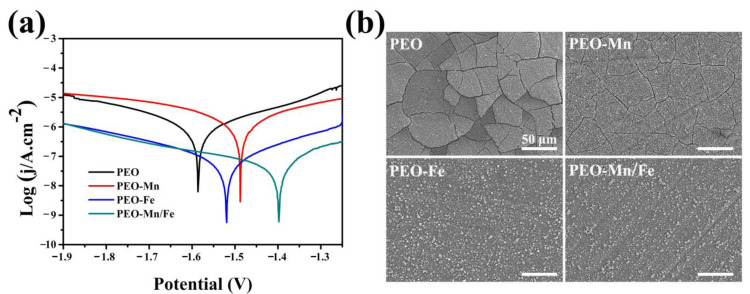
Potentiodynamic polarization curves (**a**) and corrosion morphology (**b**) of PEO, PEO–Mn, PEO–Fe, and PEO–Mn/Fe samples.

The corrosion resistance of the films was evaluated using electrochemical and immersion tests. As shown in Figure 4a, although the PEO–Mn sample exhibited a higher self-corrosion potential than did the PEO sample, their self-corrosion currents were at a similar level. However, the self-corrosion currents of PEO–Fe and PEO–Mn/Fe were significantly lower than those of the PEO and PEO–Mn samples, suggesting better corrosion resistance. Notably, the PEO–Mn/Fe sample exhibited the highest self-corrosion potential, revealing that it was most difficult to corrode. Figure 4b shows the corrosion morphology of various samples after immersion in α-MEM for 7 days. Numerous cracks were observed on the surface of the PEO sample. After MnOOH coating, the cracks were still present on the PEO–Mn surface; however, they were narrower than those on the PEO surface. This might be because the MnOOH layer on the PEO coating is too thin to prevent the corrosive fluid from permeating into the PEO layer (Figure 1a and Figure S2). Interestingly, no cracks were observed on the PEO–Fe and PEO–Mn/Fe samples, indicating the favorable corrosion resistance of the films. As shown in Figure 1a, a layer of nanosheet-like structures completely covered the PEO layer in both the groups, which could be sufficient to prevent the contact between the liquid and the PEO layer, and thus greatly improved the corrosion resistance of the samples. On the other hand, oxyhydroxide is the precursor of layered double hydroxide (LDHs) and would gradually transfer to LDHs in fluid [28]. LDHs are widely considered to be biodegradable materials for biomedical applications [32]. Therefore, the as-prepared films on the Mg alloy would not inhibit the advantage of the biodegradable ability of the magnesium alloy implants.

**Figure 5:**
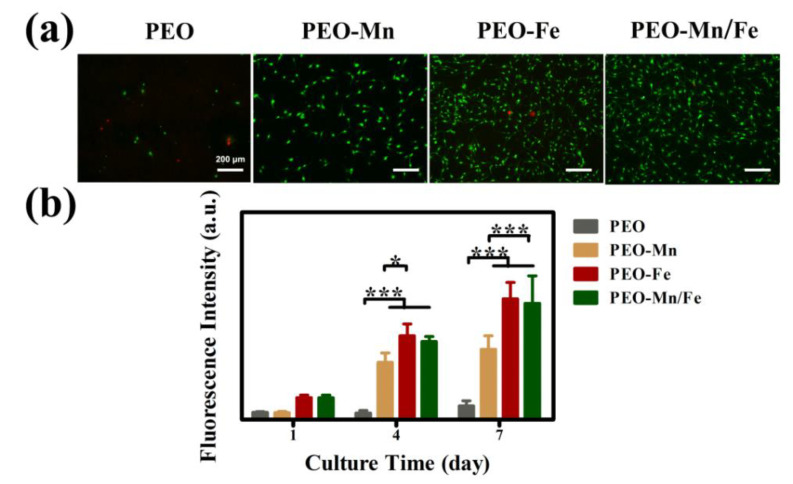
Live/dead staining (**a**) and cell proliferation (**b**) of C3H10T1/2 cells cultured on PEO, PEO–Mn, PEO–Fe, and PEO–Mn/Fe sample surfaces. (*: *p* < 0.05; ***: *p* < 0.001, and n = 4).

Cytocompatibility of the samples was evaluated by culturing C3H10T1/2 cells directly on sample surfaces. Figure 5a shows the live/dead staining results. Only a few living cells were observed on the PEO sample surface. After modifying the PEO with a MnOOH layer, more living cells were detected. Significantly larger numbers of living cells and more connected pseudopods were observed on the PEO–Fe and PEO–Mn/Fe samples than on the PEO–Mn sample. The Alamar Blue results are presented in Figure 5b. The PEO–Fe and PEO–Mn/Fe samples showed the highest proliferation rate among the four groups, consistent with the live/dead staining results. It should be noted that there were no significant differences for the live/dead staining and cell proliferation results between the PEO–Fe and PEO–Mn/Fe samples. These data suggest that the FeOOH film greatly improved the cytocompatibility of the substrate.

**Figure 6:**
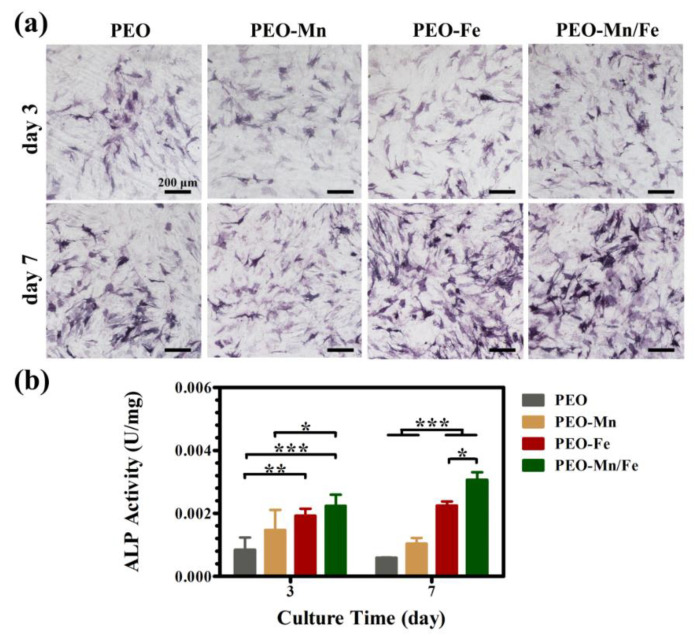
ALP activity in C3H10T1/2 cells cultured in PEO, PEO–Mn, PEO–Fe, and PEO–Mn/Fe extracts (**a**) and corresponding quantitative results (**b**). (*: *p* < 0.05; **: *p* < 0.01; ***: *p* < 0.001, and n = 4).

**Figure 7:**
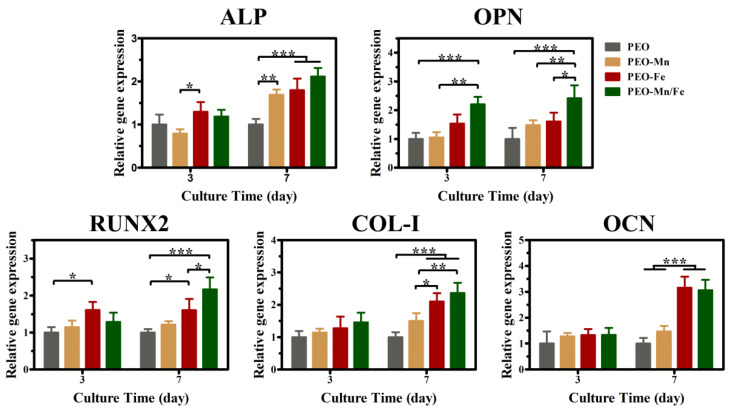
The expression of bone-related genes in C3H10T1/2 cells cultured in PEO, PEO–Mn, and PEO–Fe extracts. (*: *p* < 0.05; **: *p* < 0.01; ***: *p* < 0.001, and n = 4).

Because the cells cultured on sample surfaces showed poor viability, we used extracts to evaluate the osteogenesis induction ability of various groups, according to the ISO 10993-5 standard [33]. The ALP staining and corresponding quantitative results are shown in Figure 6a and b, respectively. The PEO–Fe and PEO–Mn/Fe groups exhibited higher ALP activity than did the other two groups at both time points. In particular, on day 7, the PEO–Mn/Fe sample exhibited the highest ALP activity. At the molecular level, the PEO–Mn/Fe group showed the highest OPN gene expression when cultured for 3 days (Figure 7). Intriguingly, after extending the culture time to 7 days, ALP, OPN, RUNX2, and COL-I expression was highest in cells cultured in the PEO–Mn/Fe extract. On day 7, OCN expression was similar for the PEO–Fe and PEO–Mn/Fe groups, but was still significantly higher than that in the other two groups. Combining the results of ALP activity and the gene expression analyses, it can be concluded that the PEO–Mn/Fe sample is most favorable for the osteogenic differentiation of bone stem cells.

**Figure 8:**
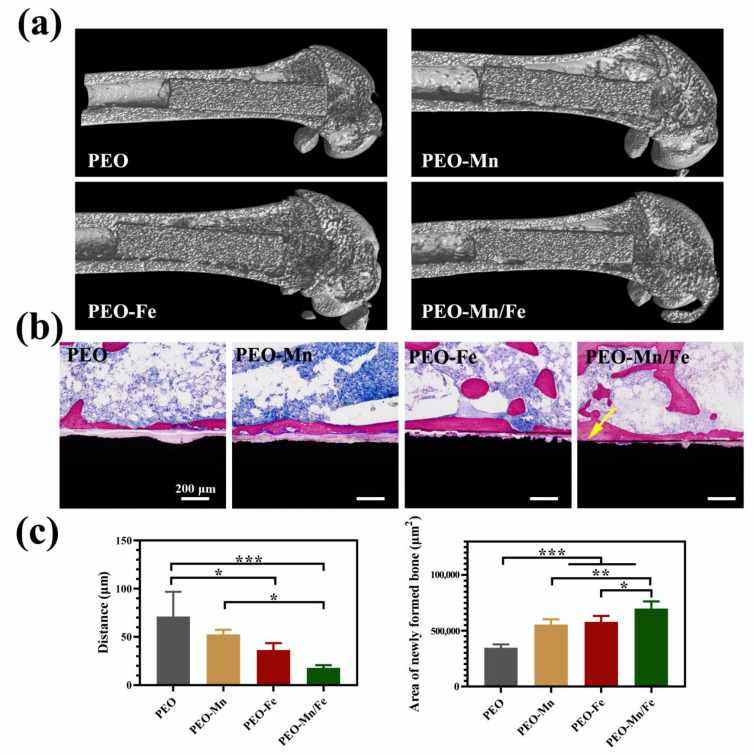
Representative Micro-CT images (**a**) and VG staining (**b**) of PEO, PEO–Mn, and PEO–Fe implants after 8 weeks of implantation. The quantitative analysis of gap distance between newly formed bone and the implants, and the area of newly formed surrounding the implants (**c**). (*: *p* < 0.05; **: *p* < 0.01; ***: *p* < 0.001). The yellow arrow in b indicates the closely adhesion between the newly formed bone and the implant.

To further investigate in vivo osteogenesis ability, all the samples were implanted in rat femurs for 8 weeks. The Micro-CT results shown in Figure 8a suggest that the structures of all femurs were normal and no bone resorption or osteonecrosis was present. The collected femurs were stained with VG solution and the results are shown in Figure 8b and the corresponding quantitative analysis is shown in Figure 8c. Large gaps between the newly formed bone and the implants were observed for the PEO and PEO–Mn groups. Notably, the narrowest gap was observed for the PEO–Mn/Fe group; additionally some newly formed bone closely adhered to the implant surface (indicated by the yellow arrow). Moreover, the largest amount of newly formed bone was observed in the PEO–Mn/Fe group. This suggested that the PEO–Mn/Fe implant was the most favorable for bone regeneration and osteointegration.

The PEO–Mn/Fe sample showed the best osteogenesis performance owing to its corrosion resistance being the best and its sustained release of Mg, Fe, and Mn ions. On the one hand, the corrosion products H_2_ and OH^-^ hugely decreased with improved corrosion resistance, thus reducing the damage to the bone remodeling process. On the other hand, all metal ions mentioned above are bioactive and essential for new bone formation. The Mg ions released from Mg implants upregulate calcitonin gene–related peptide (CGRP) and then promote osteogenic differentiation of bone stem cells [34]. Fe is involved in vitamin D metabolism and collagen synthesis, thus influencing the bone formation process [35,36]. Mn can bind to integrin and trigger integrin-mediated signaling cascades to enhance osteogenesis process [37,38]; additionally, it participates in the synthesis of chondroitin sulfate and glycosyltransferases, which play critical roles in the formation of skeletal and cartilage matrices [39]. Furthermore, there is evidence that these bioactive ions have synergistic effects on the improvement of bone regeneration [40]. Therefore, the PEO–Mn/Fe implant possessed the best bone regeneration capability was expected.

## 4. Conclusions

In conclusion, the current study successfully fabricated a duplex film with an inner MnOOH layer and an outer FeOOH layer on PEO-coated Mg alloy. The oxyhydroxide film completely sealed the pores on the PEO surface. Therefore, it inhibited the occurrence and development of cracks on the PEO layer. The modified sample exhibited improved osteogenesis induction ability in vitro and enhanced bone regeneration in vivo, owing to its better corrosion resistance and sustained release of bioactive ions, including Mg, Fe, and Mn. The novel design and fabricated oxyhydroxide-modified PEO film formed on Mg alloy shows promising potential for orthopedic applications.

## Data Availability

All raw data from the characterizations are available from the corresponding author upon request.

## Supplementary Materials

The following supporting information can be downloaded at: https://www.mdpi.com/article/10.3390/jfb13020050/s1, Figure S1: Representative optical images of PEO, PEO–Mn, PEO–Fe, and PEO–Mn/Fe samples; Figure S2: Cross-sectional images of PEO, PEO–Mn, PEO–Fe, and PEO–Mn/Fe samples; Table S1: Primer sequences of the osteogenesis-related genes used in this study.
