# Supplementary material for: Oxyhydroxide-Coated PEO–Treated Mg Alloy for Enhanced Corrosion Resistance and Bone Regeneration
Source: J Funct Biomater. 2022 May 1;13(2):50. doi: 10.3390/jfb13020050 (PMC9149893; doi:10.3390/jfb13020050)
Supplement: Supplementary file 1 [file jfb-13-00050-s001.zip › jfb-1666003-supplementary.pdf]

Supporting Information

Oxyhydroxide coated PEO-treated Mg alloy for enhanced corrosion resistance and bone regeneration

Juning Xie[1,2,#], Shi Cheng[2,#], Guoqing Zhong[2,3], Ruixiang Zhou[2,4], Chi Zhang[2], Yue He[1,2], Yu Zhang[2,1,*], Feng Peng [2,*]

[1] *School of medicine, South China university of Technology, Guangzhou 510006, China*

[2] *Medical Research Center, Department of Orthopedics, Guangdong Provincial People's Hospital, Guangdong Academy of Medical Sciences, 510080, China*

[3] *Shantou University Medical College, Shantou 515041, China*

[4] *Jinzhou Medical University, Jinzhou, 121001, Liaoning, China*

# Juning Xie and Shi Cheng contribute equally to this work.

***Correspondence: zhangyu@gdph.org.cn (Y.Z.) pengfeng@gdph.org.cn (F.P.)**

Table S1. Primer sequences of the osteogenesis-related genes used in this study.

Genes	Forward sequence	Reverse sequence
GAPDH (rat)	AAGGTCGGTGTGAACGGATTTG	TGTAGTTGAGGTCAATGAAGGGGTC
ALP	GCACAACATCAAGGACATCG	TCAGTTCTGTTCTTGGGGTACAT
RUNX2	TGTGTGCCTCCAACCTGTGT	CTTTCCCCCTCAATTTGTGTCA
OCN	ATAGACTCCGGCGCTACCTC	CCAGGGGATCTGGGTAGG
COL-I	GAAGACCTGGCGAGAGAGGA	TCAATCCATCCAGACCGTTG
OPN	CCAGCCAAGGACCAACTACA	AGTGTTTGCTGTAATGCGCC

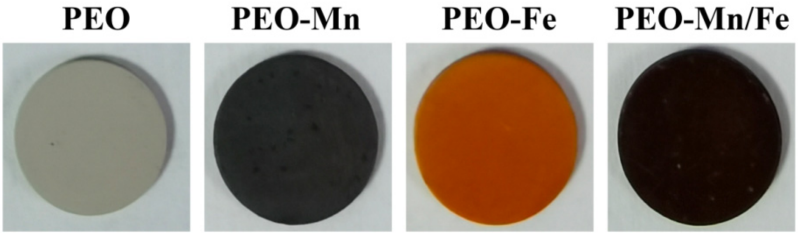

Figure S1. Representative optical images of PEO, PEO-Mn, PEO-Fe, and PEO-Mn/Fe samples.

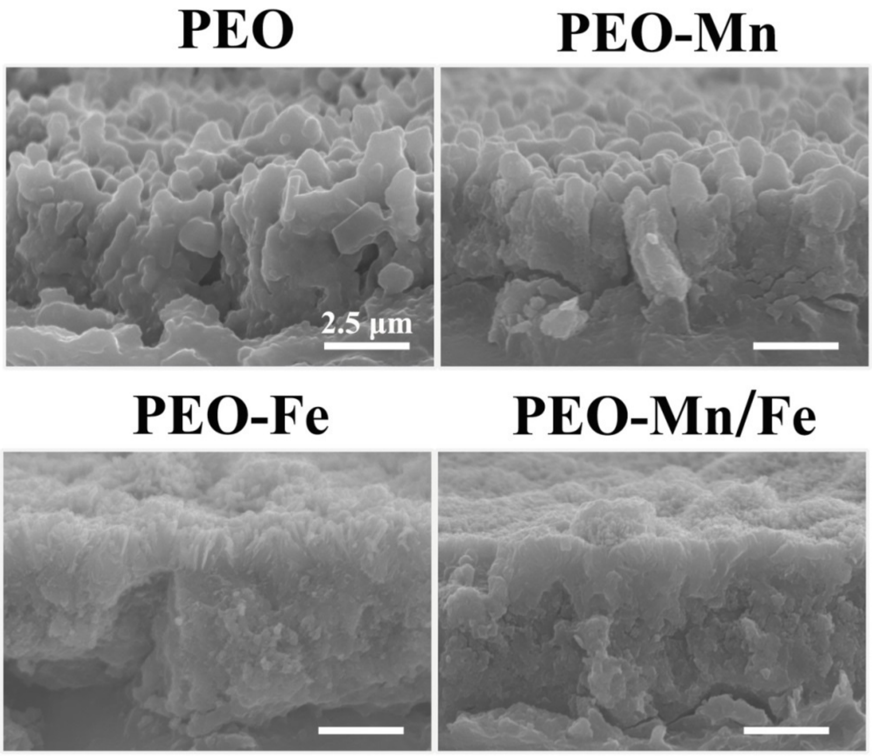

Figure S2. Cross-section images of PEO, PEO-Mn, PEO-Fe, and PEO-Mn/Fe samples.
